# A novel method to study contact inhibition of locomotion using micropatterned substrates

**DOI:** 10.1242/bio.20135504

**Published:** 2013-07-12

**Authors:** Elena Scarpa, Alice Roycroft, Eric Theveneau, Emmanuel Terriac, Matthieu Piel, Roberto Mayor

**Affiliations:** 1 Department of Cell and Developmental Biology, University College London, Gower Street, London WC1E 6BT, UK; 2 Institut Curie, CNRS UMR144, 26 rue d'Ulm, 75248 Paris Cedex 05, France

**Keywords:** Contact inhibition of locomotion, Neural crest, Micropatterned fibronectin substrates

## Abstract

The concept of contact inhibition of locomotion (CIL) describes the ability of a cell to change the direction of its movement after contact with another cell. It has been shown to be responsible for physiological and developmental processes such as wound healing, macrophage dispersion and neural crest cell migration; whereas its loss facilitates cancer cell invasion and metastatic dissemination. Different assays have been developed to analyze CIL in tissue culture models. However, these methods have several caveats. Collisions happen at low frequency between freely migrating cells and the orientation of the cells at the time of contact is not predictable. Moreover, the computational analysis required by these assays is often complicated and it retains a certain degree of discretion. Here, we show that confinement of neural crest cell migration on a single dimension by using a micropatterned substrate allows standardized and predictable cell–cell collision. CIL can thus easily be quantified by direct measurement of simple cellular parameters such as the distance between nuclei after collision. We tested some of the signaling pathways previously identified as involved in CIL, such as small GTPases and non-canonical Wnt signaling, using this new method for CIL analysis. The restricted directionality of migration of cells in lines is a powerful strategy to obtain higher predictability and higher efficiency of the CIL response upon cell–cell collisions.

## Introduction

More than five decades ago, Abercrombie and Heaysman found that the direction of migration of fibroblasts cultured in vitro was affected by their interaction with other cells (Abercrombie and Heaysman, 1953). The process was named contact inhibition of locomotion (CIL) and it was proposed as the main force driving wound healing of epithelia (Abercrombie, 1979; Abercrombie and Ambrose, 1962). CIL is defined as the ability of a cell to change the direction of its movement after contact with another cell. It consists of a stereotyped sequence of steps: (i) cell–cell contact, (ii) inhibition of membrane protrusions at the site of contact, (iii) repolarization through generation of a new protrusion away from the site of cell contact and (iv) migration in the direction of the new protrusion (Mayor and Carmona-Fontaine, 2010). The potential importance of this idea became immediately apparent when it was observed that malignant mesenchymal cells showed a reduced CIL response, being able to invade fibroblast cultures in what was compared to invasive metastasis (Abercrombie, 1979; Abercrombie and Ambrose, 1962; Abercrombie and Heaysman, 1954a). More recently, Eph-Ephrin signaling was shown to be important to regulate the invasiveness of prostate cancer cells towards stromal fibroblast via an inhibition of the CIL response in the malignant cells (Astin et al., 2010). Furthermore, the fundamental relevance of CIL in guiding complex migratory phenomena during embryonic development has been demonstrated in vivo for neural crest (NC) cells and macrophages (Carmona-Fontaine et al., 2008; Stramer et al., 2010).

CIL prevents the formation of protrusions between cells. Therefore, when cells are at high cell density only the cells with a free edge can produce lamellipodia whereas cells surrounded by other cells can only generate smaller transient protrusions. As a consequence of this behavior, cells exhibiting CIL do not crawl over their neighbours leading to monolayer formation in groups and to scattering in single cells. Furthermore, when two cell clusters exhibiting CIL-like behavior are juxtaposed, they will tend to remain separated rather than invading each other (Carmona-Fontaine et al., 2008).

**Fig. 1.**
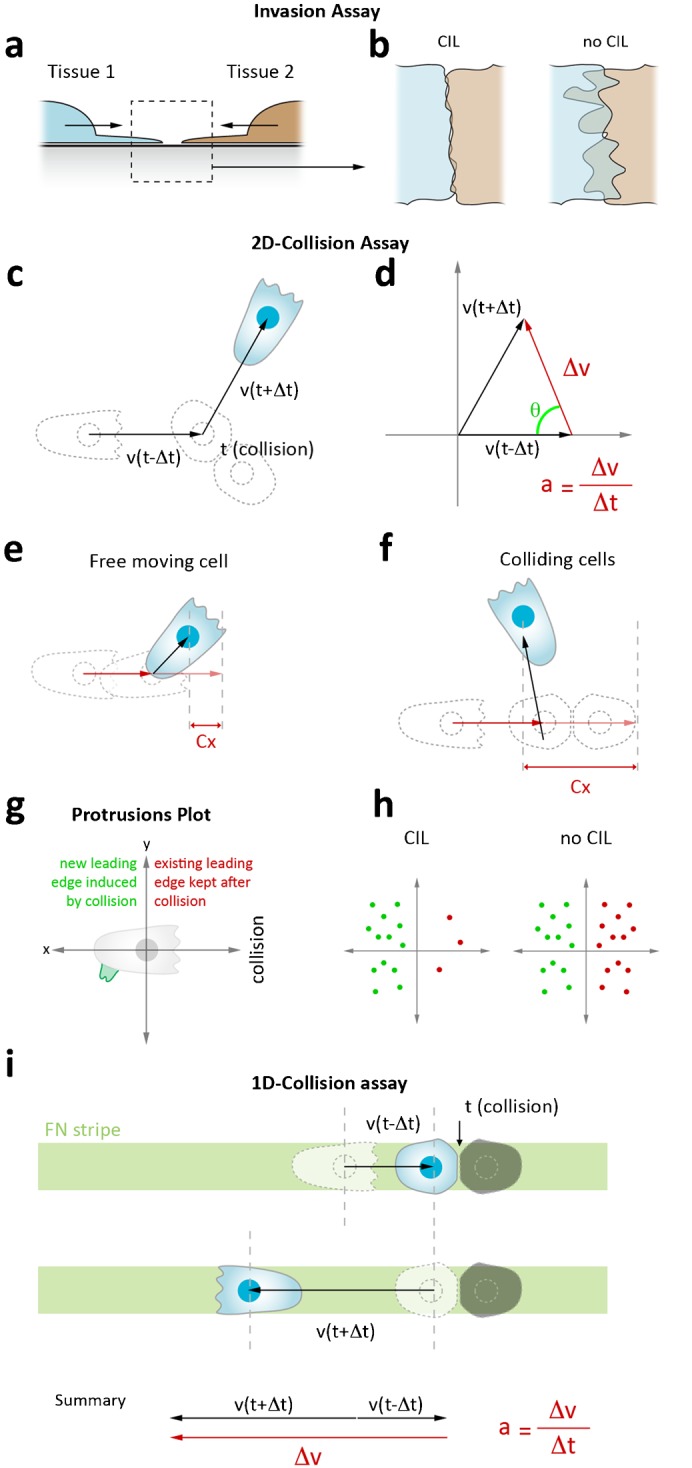
Methods to analyze contact inhibition of locomotion in tissues and cells cultured on 2D and 1D-substrates. (**a**) Invasion assay: two differentially labeled tissue explants are placed in close proximity. (**b**) If the tissues undergo CIL a sharp boundary is established between the two cell populations. In absence of CIL the tissues invade each other. This amount of overlap can be measured and used as a quantification of CIL. (**c**) 2D-collision assay: a cell colliding with another one deviates from its trajectory if exhibit CIL. (**d**) The acceleration vector α and its associated angle θ can be calculated via cell tracking. (**e**,**f**) Analysis of CIL via calculation of the acceleration index. Cx represents the difference between how far the cell has progressed in the direction of migration and how far it would have gone had there been no collision. (**g**) Protrusion plot analysis of CIL. New leading edges formed away from contact upon collision are plotted in green, existing leading edges that are not affected by the contact are plotted in red. (**h**) Upon CIL, cells preferentially form new leading edge away from contact while collapsing pre-existing protrusions at the site of contact. This results in a polarized plot with most protrusions located away from the contact. In absence of CIL the plots are symmetrical indicating that the cells have not responded to the contact. (**i**) 1D-collision assay. Cells are confined to migrate on a straight line. Colliding cells can only make head-to-head collisions and, when undergoing CIL, cells are forced to change direction at a 180° angle to move away from each other. Thus, this method abolishes the need to measure the angle between the original direction and the new direction after repolarization. The acceleration α can be calculated via cell tracking.

Since its discovery in 1953, several assays have been developed to identify, analyze and quantify CIL as a biological phenomenon. The initial observations made by Abercrombie and Heaysman were obtained by analyzing the cell behavior in the area between two embryonic chick heart explants: where the two explants encounter, the fibroblasts do not clump on top of each other. Instead, they halt their migration or disperse elsewhere (Abercrombie and Heaysman, 1954b). A similar strategy to analyze CIL behavior among group of cells has been developed for cultured Xenopus neural crest cell explants (Carmona-Fontaine et al., 2008). In invasion assays, two differently labeled pieces of NC tissue are plated adjacent to each other (Fig. 1a). Over time, the explants will tend to spread and form a monolayer thereby contacting each other. When two cell populations show reciprocal CIL they collapse their protrusions at the sites of cell–cell contact therefore remaining separated. If at least one of the explants fails to display CIL, it will invade the other tissue thus leading to an extensive overlap of the two populations (Fig. 1b). Invasion assays proved useful to functionally identify molecules involved in CIL signaling (Carmona-Fontaine et al., 2008; Theveneau et al., 2010). However, they require labeling each explant with differential markers, the use of whole tissue explants and are imaged at low magnification, thus not allowing fine dissection of the CIL phenomenon at the cellular level.

Therefore, several assays using dissociated cultured cells have been devised. In a simple collision occurring on a 2D substrate where cells can freely move, it is possible to measure cell velocity before and after collisions (Fig. 1c). CIL leads to an arrest of migration followed by a change in velocity and a consequent acceleration when cells move away from each other (Abercrombie and Heaysman, 1953). However, variation of velocity over time can occur by chance and to conclude about CIL, such changes must occur upon cell–cell collisions. Thus, the angle between the direction of migration before and after contact, which represent the repolarization owing to CIL, has to be measured as well (Fig. 1d). Statistical analysis of the distribution of the angles formed by the position of a cell before and after contact demonstrates that these changes are not stochastic but are strongly biased in the opposite direction to the collision (Carmona-Fontaine et al., 2008).

Another means through which CIL is quantified (Astin et al., 2010; Paddock and Dunn, 1986) is by comparing contact acceleration indices (Cx) of free-moving cells and colliding cells. Cells are tracked before and after collision, and analysis of vectors is used to indicate how a cell's migration path deviates from a straight line after collision (Fig. 1e,f). This deviation is represented as a contact acceleration index (Cx) which represents the difference between how far the cell has progressed in the direction of migration and how far it would have gone had there been no collision.

Finally, as CIL induces formation of new protrusions away from the cell–cell contact, it can be quantified by plotting the distribution of persisting versus de novo protrusions after contact (Fig. 1g,h) (Kadir et al., 2011).

However, there are limitations to these measurements when culturing cells on 2D substrates. Cells can collide at any incoming angle. In fact, Abercrombie and colleagues showed that a stronger response to the contact is observed with leading edge to leading edge (head-to-head) collisions compared to a leading edge to cell body (head-to-side) (Abercrombie and Dunn, 1975). In addition, cells do not instantly separate upon collision and can rotate while in contact, making the measurement of angle difficult. Analysis of Cx is based on projection of the velocity vectors which get rid of the angles all together. Since cells colliding at various angles can show great differences in their response critical information is lost in this analysis. Protrusion plots are a fine description of the change of cell polarity after contact but they do not take into account any motility features of the colliding cells, such as velocity or acceleration before and after contact, nor the angles. Finally, because the cells are randomly migrating in a 2D environment, the frequency of cell–cell collisions events is low and the predictability of the collisions is scarce, making the collision assays in dissociated cells inefficient. Moreover, the computational analysis that follows image acquisition required by these assays can be complicated and often requires an intermediate step of cell tracking (as for assays shown in Fig. 1c–f), and the extraction of indirect parameters (acceleration, Cx acceleration index) to ascertain CIL.

Here, we develop a novel 1D collision assay (Fig. 1i). Cell migration is confined on straight fibronectin lines obtained by microcontact printing-based micropatterning. Restriction of their movements on a single dimension forces cells to collide head-to-head. Collisions are followed by a complete repolarization and migration at a fixed 180° angle, therefore standardizing the CIL response by eliminating the variability associated to the redirection angle.

## Results and Discussion

### A new way to look at contact inhibition of locomotion: 1D collision assays on micropatterned substrates

**Fig. 2.**
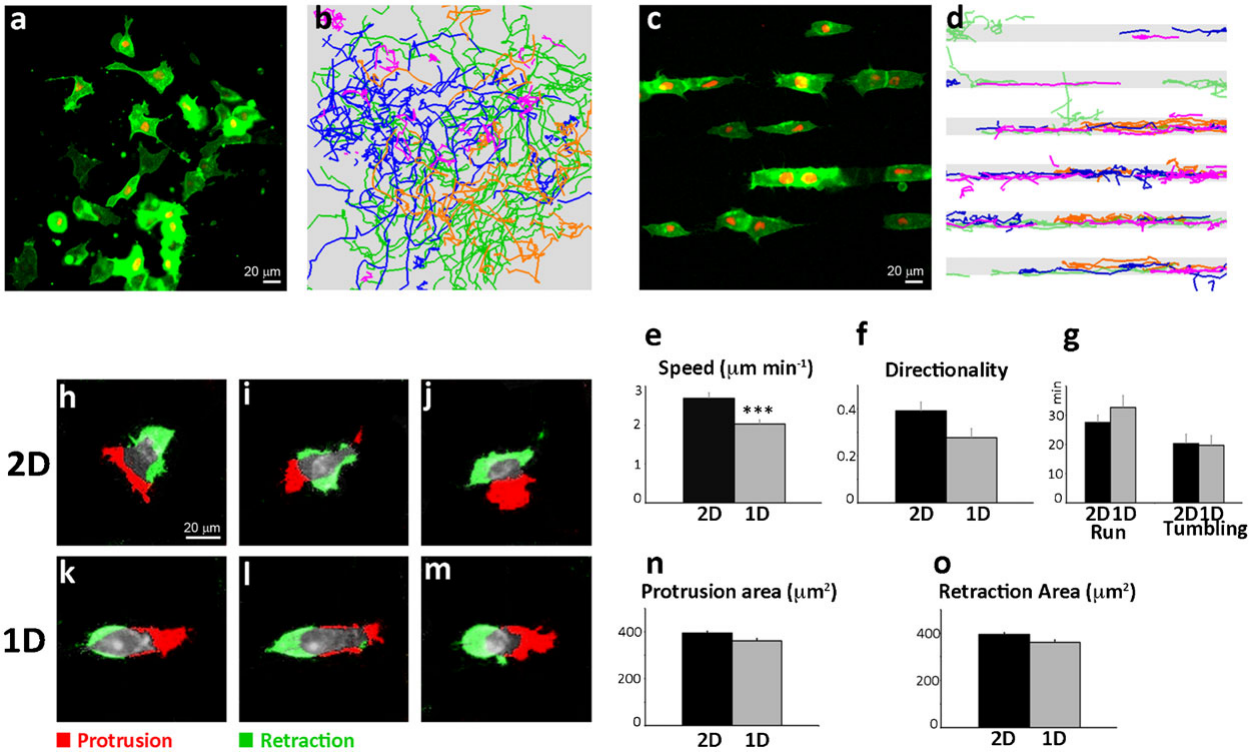
Overall dynamics of neural crest cell migration is not affected by 1D-cultures. (**a**) Still photograph from a time-lapse movie showing dissociated NC cells migrating in all directions on a 2D fibronectin substrate. (**b**) Cell tracking analysis show that NC cells migrate randomly (4 representative batches have been overlapped). (**c**) NC cell migration on 1D fibronectin lines. (**d**) Cell tracks reveal spatial restrictions imposed by the pattern (4 representative batches have been overlapped). (**e**) Average velocity on 2D and 1D matrices (***P<0.001). (**f**) Directionality of migration on 2D and 1D cultures. (**g**) Average time single migrating cells spend on “run” or “tumble” mode in 2D and 1D conditions. (**h–m**) Image subtraction analysis on single migrating cells in 2D (h–j) and 1D (k–m) cell cultures. Protrusions and retractions are color-coded in red and green respectively. (**n**) Average protrusive area for 2D and 1D substrates. (**o**) Average retraction area on 2D or 1D cultures. All error bars represent the standard error of the mean. Scale bars: 20 µm.

Micropatterning allows the control of cell adhesion geometry on a surface, and proved an inspiring technique for several questions in cell biology. It allowed important biological findings, in the fields of apoptosis (Chen et al., 1997), control of cell–cell architecture (Théry et al., 2006a), cell internal organization (Théry et al., 2006b), and division axis (Théry et al., 2005). Here, we adapted a micropatterning strategy (Théry and Piel, 2009) to standardize the analysis of CIL. Xenopus neural crest cells are cultured on 22-µm wide straight fibronectin lines. The width of the lane was chosen according to the size of neural crest cells, to avoid major effects on cell motility and cell polarity as described for narrower lanes (Doyle et al., 2009). The migratory features of Xenopus NCCs under 2D (Fig. 2a,b; supplementary material Movie 1) and 1D (Fig. 2c,d; supplementary material Movie 1) culture conditions were compared. Non-colliding single cells were tracked over time and their overall behavior analyzed. We found the speed (Fig. 2e) and directionality (Fig. 2f) to be slightly reduced on 1D-substrates. This might be due to the constraint imposed by the 1D-culture. While on a 2D-substrate cells can move in any direction; on a line, cells are forced either to keep walking forward or to completely repolarize. In addition, the repolarization process is not instantaneous. Thus, space constraint on 1D-culture is likely to account for the observed reduction of both velocity and directionality.

Migration of individual NCC cells has been characterized by an alternation between two phases: run and tumble (Theveneau et al., 2010). “Run” corresponds to a phase of directional migration, while “tumble” is a reorientation phase characterized by collapse of protrusions and by a series of small, randomly oriented movements, with no net migration. Alternation between run and tumbling phases reflects the intrinsic tendency of NCCs to change direction. Importantly, this cycle of run and tumble phases, measured as described (Theveneau et al., 2010), is not altered by the 1D-substrates (Fig. 2g). To further characterize the migratory behavior of single cells in 1D-cultures, we performed image subtraction analysis that allows to distinguish and to quantify the protrusive activity at the leading edge and the trailing edge retraction of a migrating cell (Fig. 2h–m). We measured the average membrane protrusion area (Fig. 2n) and the average trailing edge retracting areas (Fig. 2o), which are not affected by spatial restriction of migration on 1D lines. Overall, our comparative analysis of NC cell migration on 2D versus 1D-cultures does not reveal any major differences in cell behavior and migratory abilities.

### Analysis of collisions in 1D-cultures

**Fig. 3.**
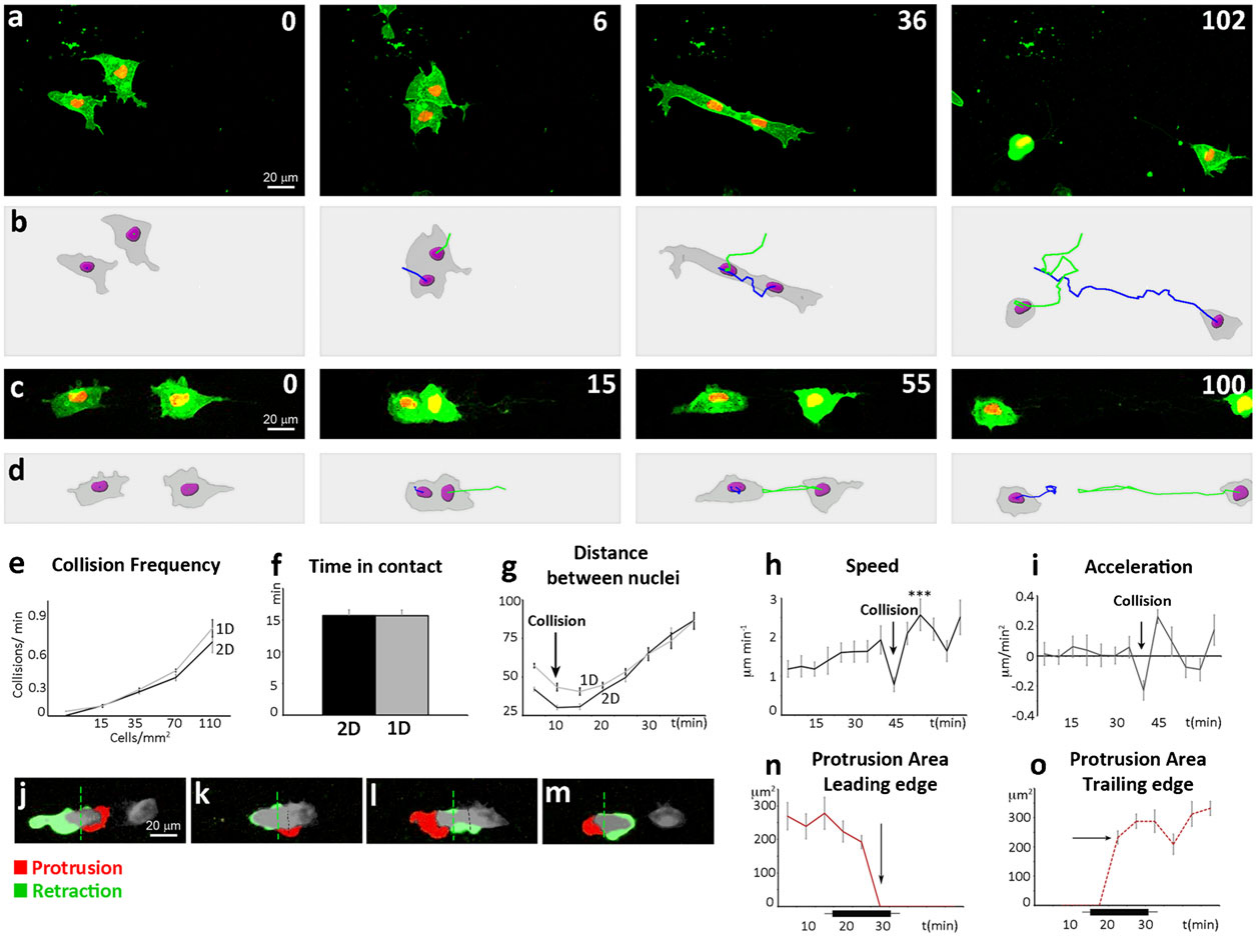
Migration on 1D-substrates does not affect contact inhibition of locomotion. (**a**) 2D collision. (**b**) Tracks of the cells shown in panel A. Both cells repolarize upon collision making a protrusion away from the contact (third frame). Since cells can rotate while in contact their overall path does not seem affected by the collision. A typical CIL analysis measuring the angle of acceleration would wrongly categorize these cells as non-responding. (**c**) 1D collision. (**d**) Tracks of the cells shown in panel C. Note that both cells move away from each other at a sharp 180° angle. (**e**) Frequency of collisions per time frame plotted against cell density. (**f**) Average time colliding cells spend in contact. (**g**) Over time variation of the average distance between nuclei of colliding cells. The first timepoint of collision is indicated by the black arrow. (**h**) Speed of colliding cells on 1D lines is plotted against time. The first timepoint of collision is indicated by the black arrow (***P<0.001). (**i**) Acceleration of colliding cells on 1D lines is plotted against time. The first timepoint of collision is indicated by the black arrow. (**j–m**) Image subtraction analysis of a NC–NC collision. Protrusive activity is represented in red, retraction is represented in green. For quantitative analysis, each colliding cell was subdivided (green dashed line) in a leading edge side and a trailing edge side. (**n**) Leading edge protrusion area was calculated over time upon NC–NC collisions. The black bar under the *x*-axis represent the average time the cells spent in contact. Arrow indicates the timepoint at which the protrusion at the cell–cell contact site collapses. (**o**) Trailing edge protrusion area was calculated over time upon NC–NC collisions. The black bar under the *x*-axis represent the average time the cells spent in contact. Arrow indicates the timepoint at which a new protrusion is formed away from cell–cell contact. Scale bars: 20 µm.

We then compared collisions occurring on 2D (Fig. 3a,b) and 1D-substrates (Fig. 3c,d). First, we asked whether the frequency of collision would improve by plating cells on a 1D-substrate. Frequency of collisions increases proportionally to the cell density in an exponential manner in both 2D and 1D-cultures but no significant difference was observed (Fig. 3e). We then looked at the time two cells spend in contact and how the distance between them evolves over time during CIL, using the cell nuclei as references. Importantly, the average time cells spend in contact with each other is unchanged (Fig. 3f), suggesting that CIL occurs with comparable dynamics on 2D and 1D-cultures. During collision, the distance between the nuclei drops at the time point of physical contact and remains constant as long as the cells stay together (Fig. 3g). The distance then linearly increases as the cells move away. We empirically determined that 30 minutes after collision most cells have migrated away from each other and distance between cells at this time point can thus be used as an easy readout of CIL.

Data extracted from cell tracking analysis of cells on 1D-substrates revealed a similar behavior upon CIL to those previously described for cells in 2D-conditions (Carmona-Fontaine et al., 2008). A drop in cell speed at the time of contact (Fig. 3h, arrow) is followed by a sudden increase (Fig. 3h). This can be shown as a deceleration upon contact (Fig. 3i, arrow) followed by an acceleration (Fig. 3i). Importantly, this acceleration is coupled with a repolarization in the opposite direction. This can be assessed by monitoring protrusion/retraction dynamics over time. To do so, growing and retracting regions of the cells are color-coded by image subtraction (Fig. 3j–m; see Materials and Methods for details). Before collision, the cell extends a protrusion at its leading edge (Fig. 3j, red) oriented towards the other cell. Upon collision (Fig. 3k), the protrusion collapses, and a new protrusion is extended on the former trailing edge of the cell (Fig. 3l). This represents a complete switch of cell polarity. The cells eventually separate (Fig. 3m). The average protrusion and retraction areas were measured over time (Fig. 3n,o). Interestingly, the switch of polarity occurs immediately after the two cells make contact. The initial protrusion collapses at the time of contact (Fig. 3n, arrow) and a new protrusion is created at the opposite side of the cell (Fig. 3o, arrow).

To summarize, CIL occurs with similar frequency and comparable timing in 2D and 1D-cultures. Critically, the duration of the cell–cell contact, the dynamics of cell protrusion leading to repolarization and its consequences on cell velocity and acceleration are preserved on 1D-substrates.

### Standardization and validation of a unique parameter for assaying CIL

**Fig. 4.**
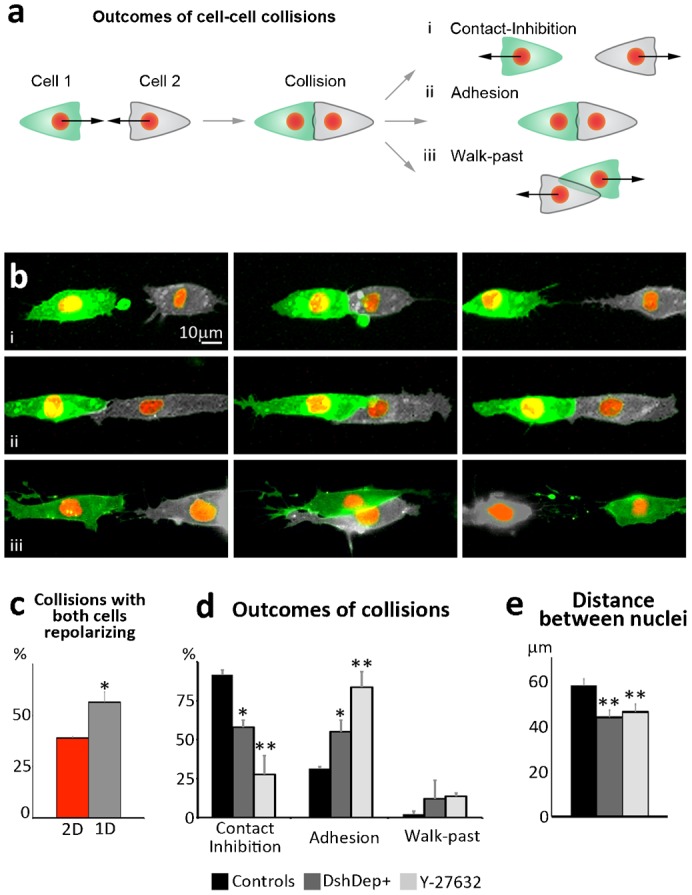
1D-substrates increase the probability of successful collision and provide a simpler readout of CIL. (**a**) Diagram showing the possible outcomes of cell–cell collisions on fibronectin lines. (**b**) Examples showing the different outcomes depicted in panel A. When CIL occurs it leads to complete repolarization of the direction of migration with cells moving away from each other (**ai**,**bi**). Alternatively, cells can fail to dissociate after contact and adhere to each other (**aii**,**bii**). Finally, cells may not react to their physical contact and walk past each other following their original path of migration (**aiii**,**biii**). (**c**) Percentage of cell–cell collisions in which both colliding cells repolarize upon contact in 2D or 1D cultures (*P<0.05). (**d**) Percentages of NC cells displaying CIL, adhesion or Walk-Past behavior in control conditions or upon Wnt/PCP inhibition (DEP+) or Rho Kinase inhibition (Y-27632). *P<0.05; **P<0.01. (**e**) Distance between nuclei of colliding cells 30 minutes after initial contact in control conditions or upon DEP+ or Y-27632 treatment (**P<0.01). Scale bar: 10 µm (b).

In a 1D collision assay, cell migration is restricted on straight lines. When cells enter in contact with one another there are three main outcomes. If cells exhibit a clear CIL response, cell–cell contact results in full repolarization and migration of the two cells away from each other (Fig. 4ai,bi; supplementary material Movie 2). If the cells do not exhibit CIL towards each other they may not dissociate the contact established upon collision and remain together (Fig. 4aii,bii; supplementary material Movie 2) or may be unaffected by their physical interaction and walk past each other (Fig. 4aiii,biii; supplementary material Movie 2). Therefore, spatial confinement of cell migration simplifies the distinction between a CIL and a non-CIL response. We then analyzed whether the quality of the collisions would be improved by the 1D culture conditions. In 2D, we defined a collision “successful” as an event where both cells efficiently repolarized upon contact with one another and moved away. Importantly, we observed that the percentage of successful collisions was significantly increased on 1D substrates (Fig. 4c). Early observations by Abercrombie and Dunn on chick heart fibroblast indicated that the cells undergo a stronger repolarization response when they enter in contact with each other in a head-to-head fashion when both leading edges make a frontal contact (Abercrombie and Dunn, 1975). Since cells are forced to interact head-to-head when cultured on 1D-substrates, this might account the observed improvement of the efficiency of CIL on 1D versus 2D-cultures.

The micropatterning technique here described allows both higher predictability and higher efficiency of the CIL response in cell–cell collisions. Because all of the functional assays available so far to assess CIL require more than one quantitation step and very often include cell-tracking, we aimed at identifying a cellular parameter whose direct measurement could provide an easy readout of CIL. The distance between the nuclei of two colliding cells (Fig. 3k) appeared as an interesting candidate parameter, as it is maintained relatively constant if the cells establish a contact but increases linearly when two cells repolarize and move away. Time-lapse movies are still needed to be able to distinguish cells that are far away due to CIL or walk-past behavior but importantly cell tracking is not necessary.

To validate this method we tested some of the signaling pathways that have been described to be involved in CIL of neural crest cells (Carmona-Fontaine et al., 2008). We blocked CIL by interfering with Wnt/PCP and Rho signaling using a dominant-negative of Dishevelled (DshDEP+) (Carmona-Fontaine et al., 2008) and the ROCK inhibitor Y-27632 (Carmona-Fontaine et al., 2008; Kadir et al., 2011). Collision between control cells, DshDEP+ expressing cells or Y-27632 treated cells were analyzed qualitatively (Fig. 4d) and the distance between nuclei was measured in parallel (Fig. 4e). A significant reduction in the CIL response was observed for both treatments. Importantly, the loss of CIL was associated with a significant reduction of the distance between nuclei of colliding cells 30 minutes after the initial contact (Fig. 4e) confirming that the distance between cell nuclei at a given time point after collision is a good readout of CIL.

Here we show how a micropatterning technique can facilitate the analysis of a phenomenon of significant biological relevance such as CIL. Confinement provided by one-dimensional cultures facilitates the identification of cell–cell collisions by increasing their predictability. It improves the efficiency of CIL by forcing the cells to undergo head-to-head collisions and eliminating head-to-side collisions. Moreover, by preventing random movement and rotation during collision, 1D-cultures abolish the time-consuming steps of monitoring angles of acceleration and performing cell tracking. Furthermore, it simplifies the analysis of changes in polarity by image subtraction. Finally, simplification provided by cultures on fibronectin lines allows detection of a functional CIL response by measurement of a simple and unique parameter such as the distance between two cell nuclei.

## Materials and Methods

### Microinjections, RNAs and chemical inhibitors

Xenopus laevis embryos were obtained via in vitro fertilization after gonadotropin stimulation. Embryos were let to develop till the 8-cell stage and then microinjected into the dorsal and ventral animal blastomeres on one side of the embryo with the following mRNAs: membrane-GFP (500 pg), nuclear-RFP (500 pg), DshDEP+ (1 ng). ROCK inhibition was obtained by treating the NCCs with Y-27632 (Calbiochem) 30 µM for 2 hours before imaging. mRNA transcriptions were performed with the SP6 mMessage machine kit, Ambion.

### Neural crest culture

Xenopus cranial NC cells were dissected as described (DeSimone et al., 2005). Briefly, at stage 18, the pigmented epidermal layer is removed then NC cells are gently taken out by microdissection. Cell dissociation was performed by incubating the NC explants in Ca^2+^/Mg^2+^-free DFA medium for a few minutes before transferring them to Danilchick's culture medium.

### Microcontact printing of patterned fibronectin

PDMS stamps were prepared as described (Théry and Piel, 2009) with micropatterned lines 22-µm wide spaced by 44-µm wide intervals. Stamps were incubated with fibronectin 10 µg/ml in PBS for 1 hour at 37°C, washed three times with PBS, dried and printed manually on 60 mm plastic tissue culture dishes (Falcon) for 5 minutes. Micropatterned dishes were then incubated with BSA 0.1%/PBS for 30 minutes and washed three times with PBS before use.

### Time-lapse cinematography, cell tracking, and cell protrusion analysis

Time-lapse movies and cell tracking of migrating NC cells were performed as previously described (Carmona-Fontaine et al., 2008). Tracks were generated using ImageJ Manual Tracking plug-in and Imaris. “Run and tumble” was analyzed as described (Theveneau et al., 2010). Cell protrusions were analyzed as described (Carmona-Fontaine et al., 2008). In brief, cell protrusions were defined by the positive difference in the area of a cell between two consecutive frames. Retraction was defined as the negative difference in the area of a cell between two consecutive frames. Protrusion and retraction areas were analyzed by using the ImageJ Analyze Particle plug-in. Distance between nuclei was calculated on ImageJ. Velocity and acceleration were extracted from Imaris tracks. Statistical analyses were performed using Excel software and Prism4.

## Supplementary Material

Supplementary Material
