# A novel method to study contact inhibition of locomotion using micropatterned substrates

**DOI:** 10.1242/bio.020917

**Published:** 2016-10-15

**Authors:** Elena Scarpa, Alice Roycroft, Eric Theveneau, Emmanuel Terriac, Matthieu Piel, Roberto Mayor

There was an error published in *Biol. Open* **2**, 901-906.

In Fig. 4D, the *y*-axis scale was incorrect; the correct Fig. 4 is shown below. There are no changes to the figure legend, which is accurate. This error does not affect the conclusions of the paper.

**Fig. 4.**
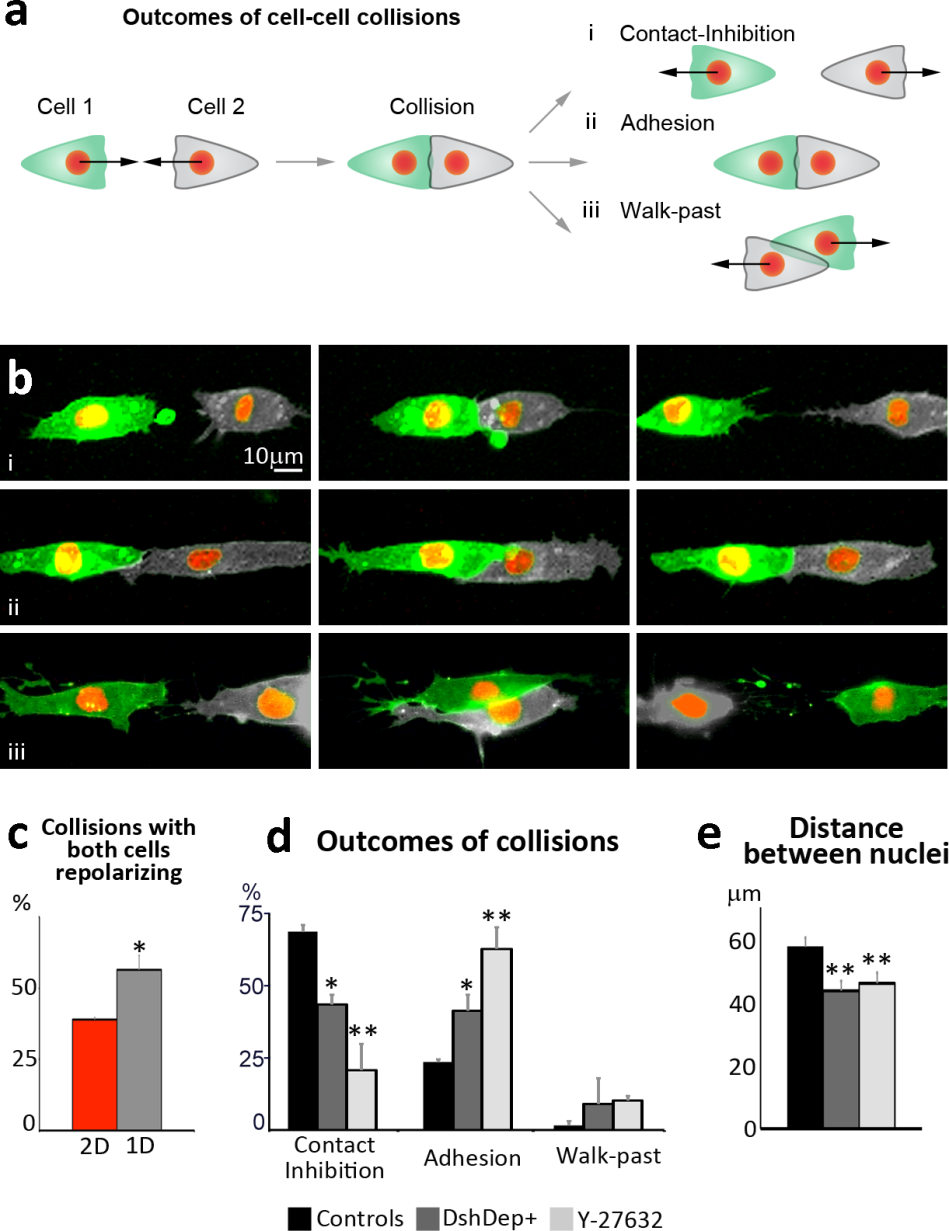
**1D-substrates increase the probability of successful collision and provide a simpler readout of CIL.** (**a**) Diagram showing the possible outcomes of cell–cell collisions on fibronectin lines. (**b**) Examples showing the different outcomes depicted in panel A. When CIL occurs it leads to complete repolarization of the direction of migration with cells moving away from each other (**ai,bi**). Alternatively, cells can fail to dissociate after contact and adhere to each other (**aii,bii**). Finally, cells may not react to their physical contact and walk past each other following their original path of migration (aiii,biii). (**c**) Percentage of cell–cell collisions in which both colliding cells repolarize upon contact in 2D or 1D cultures (*P<0.05). (**d**) Percentages of NC cells displaying CIL, adhesion or Walk-Past behavior in control conditions or upon Wnt/PCP inhibition (DEP+) or Rho Kinase inhibition (Y-27632). *P<0.05; **P<0.01. (**e**) Distance between nuclei of colliding cells 30 minutes after initial contact in control conditions or upon DEP+ or Y-27632 treatment (**P<0.01). Scale bar: 10 μm (b).

The authors apologise to the readers for any confusion that this error might have caused.

