# Supplementary material for: A novel method to study contact inhibition of locomotion using micropatterned substrates
Source: Biol Open. 2013 Jul 12;2(9):901–6. doi: 10.1242/bio.20135504 (PMC3773336; doi:10.1242/bio.20135504)
Supplement: Supplementary Material [file supp_2_9_901__index.html]

A novel method to study contact inhibition of locomotion using micropatterned substrates — Supplementary Material 

# A novel method to study contact inhibition of locomotion using micropatterned substrates

## bio.20135504 Supplementary Material

**Files in this Data Supplement:**

- Supplementary Material - Elena Scarpa et al. doi: 10.1242/bio.20135504
- Movie 1 - **Movie 1. Time-lapse movie showing a 2D collision compared with a 1D collision on fibronectin stripes.** Their respective tracks are shown below each movie.
- Movie 2 - **Movie 2. Time-lapse movie showing the possible outcome of cell–cell collisions on 1D culture.** Cells can either undergo CIL, form a cell–cell adhesion, or walk past each other.
