# Supplementary material for: A novel method to study contact inhibition of locomotion using micropatterned substrates
Source: Biol Open. 2013 Jul 12;2(9):901–6. doi: 10.1242/bio.20135504 (PMC3773336; doi:10.1242/bio.20135504)
Supplement: Supplementary Material [file supp_bio.20135504_bio.20135504-s1.pdf]

Biology Open

Supplementary Material

Elena Scarpa et al. doi: 10.1242/bio.20135504

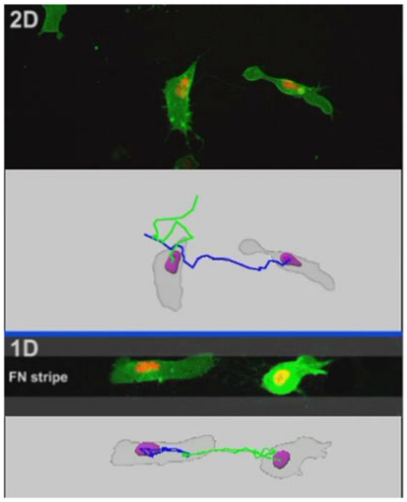

Movie 1. Time-lapse movie showing a 2D collision compared with a 1D collision on fibronectin stripes. Their respective tracks are shown below each movie.

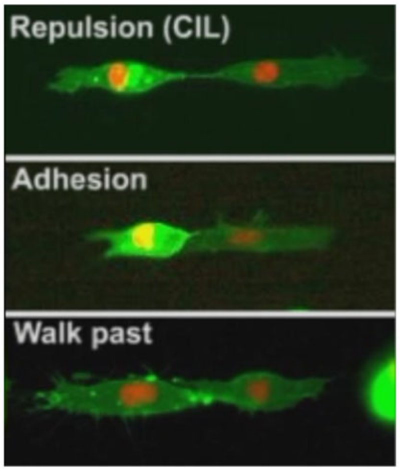

Movie 2. Time-lapse movie showing the possible outcome of cell–cell collisions on 1D culture. Cells can either undergo CIL, form a cell–cell adhesion, or walk past each other.
